# Who’s Driving? Human Cytomegalovirus, Interferon, and NFκB Signaling

**DOI:** 10.3390/v10090447

**Published:** 2018-08-21

**Authors:** Christopher M. Goodwin, Jessica H. Ciesla, Joshua Munger

**Affiliations:** Department of Biochemistry and Biophysics, University of Rochester Medical Center, Rochester, NY 14642, USA; Christopher_Goodwin@URMC.Rochester.edu (C.M.G.); Jessica_Ciesla@URMC.Rochester.edu (J.H.C.)

**Keywords:** human cytomegalovirus, HCMV, NFκB, interferon, IκB kinase (IKK), ISG

## Abstract

As essential components of the host’s innate immune response, NFκB and interferon signaling are critical determinants of the outcome of infection. Over the past 25 years, numerous Human Cytomegalovirus (HCMV) genes have been identified that antagonize or modulate the signaling of these pathways. Here we review the biology of the HCMV factors that alter NFκB and interferon signaling, including what is currently known about how these viral genes contribute to infection and persistence, as well as the major outstanding questions that remain.

## 1. Introduction

Human cytomegalovirus (HCMV) is a widespread opportunistic pathogen that causes disease in a variety of immunosuppressed populations, including the elderly, cancer patients, and AIDS patients [1,2]. HCMV infection also causes significant morbidity in transplant recipients, and it is a major cause of kidney, liver, heart and bone marrow transplant rejection [3]. HCMV infection is also a significant cause of congenital disability, as roughly 5 out of every 1000 infants born each year are infected with HCMV, and approximately 10% of that population will experience neurological symptoms [2,4,5].

The relationship between HCMV and host immunity, including recognition, priming, and the subsequent host response, is a major determinant of HCMV pathogenesis. The earliest events of this response typically involve innate immune sensing of infection in non-immune cells, induction of an anti-viral state, and secretion of anti-viral paracrine factors that both help neighboring cells resist infection as well as recruit and activate professional immune cells. Interferon and NFκB signaling are two signal transduction cascades integral to these processes that are frequently targeted by viral infection to ensure persistence. The past 25 years have seen great progress in identifying numerous HCMV viral factors that modulate these innate immune pathways. However, many questions remain about both the specific biochemical mechanisms involved as well as the contexts through which they contribute to viral pathogenesis, replication, and persistence. Given their importance to infectious outcomes, further elucidating the diverse roles of these viral innate immune modulators remains a high priority to further our understanding of viral biology, while also potentially providing fertile ground for therapeutic development.

## 2. HCMV and Interferon Signaling

Interferons (IFN) are a broad class of cytokines initially recognized for their ability to protect cells from viral infection. Anti-viral IFN signaling in response to infection is capable of inducing a variety of host cell defenses targeting various aspects of virus biology, including global inhibition of translation via activation of protein kinase R (PKR), induction of RNAse L-mediated degradation of viral RNA, production of anti-viral nitric oxide through inducible nitric oxide synthase (iNOS), depletion of tryptophan via the indoleamine 2,3 dioxygenase (IDO) pathway, and upregulation of antigen presentation via major histocompatibility (MHC) complex constituents [6,7,8,9,10]. IFN signaling is sub-classified into Type I, Type II, and Type III groups based on their specific signaling architectures and outcomes. The majority of interferon-induced anti-viral activity is dependent on the diverse activities of Type I IFNs, which in humans include IFNκ, IFNε, IFNω, IFNβ, and a host of IFNα isoforms derived from 13 separate genes [11]. The activities of Type I IFNs induce the expression of a large set of IFN-stimulated genes (ISGs) that are critical for rendering the host cell environment less permissive for viral infection. In addition to the direct effects of Type I cytokines on ISG induction, these IFNs have been implicated in coordinated innate immune effector processes including Natural Killer (NK) cell activation, dendritic cell (DC) maturation, and T-cell differentiation [12]. In contrast to the diverse signaling of Type I IFNs, Type II IFN signaling is comprised entirely of the activities of a single cytokine: IFNγ. While IFNγ is also capable of activating ISG expression, its primary function appears to center around enabling the activation, recruitment, and survival of a diverse array of immune cells [13]. The final interferon subtype, Type III, was discovered more recently. Like Type I IFN signaling, Type III IFN signaling plays an important role in host cell defense against viral infection, but it relies on a different subset of IFNs (IFN-λ1, IFN-λ2, and IFN-λ3) and a distinct, heterodimeric IL28Rα/IL10Rβ receptor to induce the expression of anti-viral ISGs. As expression of the Type III IFN receptor is limited to epithelial cell populations, Type III IFN signaling is restricted to a more niche biological context than Type I signaling [14]. HCMV gene products that interact directly with Type III signaling have yet to be described. Canonical signaling of all three IFN subtypes occurs through the activation of the JAK-STAT pathway, but each subtype coordinates with unique cellular receptors and recruits specific STAT proteins that tailor downstream responses to respective Type I, II, and III IFN signaling inputs (reviewed in [15]).

**Figure 1:**
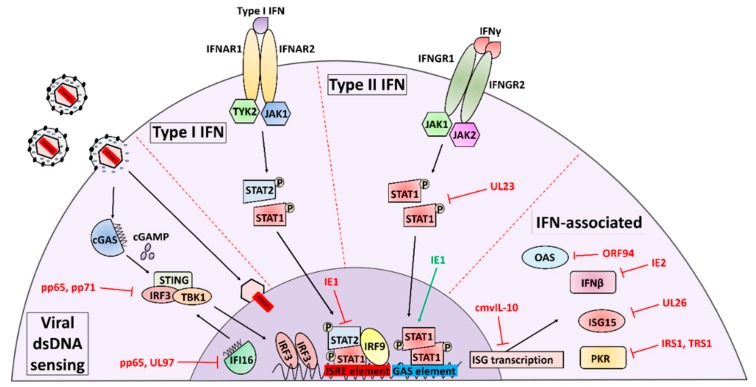
HCMV-mediated modulation of Interferon signaling.

The IFN response to HCMV infection is complex, with multiple distinct mechanisms of IFN activation and temporal peaks of IFN activity occurring over the viral life cycle. The initial interferon response to HCMV infection is triggered when the cell detects viral attachment and entry, resulting in an early induction of IFN synthesis and secretion [16]. The list of cellular sensors that detect and are activated by HCMV binding and entry continues to expand, and includes the TLR2 (toll-like receptor) and CD14 receptors that interact with viral gB and gH [17,18] as well as the intracellular dsDNA sensors Z-DNA binding protein 1 (ZBP1) [19], TLR9 [20], and cGAS [20], all of which detect the presence of the viral genome in the host cell. Seemingly in response to these cellular defenses, HCMV has evolutionarily developed a suite of IFN countermeasures that occupy a significant portion of viral coding potential (summarized in Figure 1 and reviewed in [12,21,22]).

### 2.1. The HCMV Tegument Proteins and Interferon Modulation

Given that the IFN response to infection is a swift anti-viral response set in motion by the first interactions between virion glycoproteins and cellular receptors, it is logical that HCMV encodes viral effectors that immediately antagonize these early anti-viral events, and downregulation of IFN signaling during HCMV infection is well-documented [23,24,25,26]. Many of these early viral anti-interferon factors are tegument proteins that are delivered to the cellular cytoplasm upon initial envelope fusion. One of the first of these HCMV-encoded IFN modulators described was the U_L_83-encoded tegument protein pp65, which is packaged into the virion in such abundance that it is the major constituent of the infectious particle [27]. The pp65 protein plays a major role in inhibiting both innate and adaptive immune responses to HCMV infection by interfering with antigen presentation [28] and NK cell activation [29]. HCMV pp65 was initially characterized as a Type I IFN inhibitor through early reports utilizing a U_L_83-deletion virus that induced IFN-associated transcriptional patterns during infection. Varying hypotheses of pp65’s mechanistic function emerged as some groups reported that pp65 was sufficient to block activation and nuclear localization of IRF3, a primary mediator of Type I IFN signaling [30], while others reported no change in IRF3 activity but observed that pp65 loss significantly increased IRF1 and NFκB nuclear localization [31]. Subsequent studies suggested that the pp65 deletion mutant virus exhibited impaired expression of other important HCMV genes, including the immediate-early protein 2 (IE2), and found that a less-disruptive mutation of the pp65 ORF maintained viral inhibition of Type I IFN signaling during infection [32], indicating that pp65 may be more dispensable for inhibition of IFN signaling than was initially presumed.

The question of how pp65 contributes to HCMV-mediated inhibition of IFN activity is largely still unresolved. While some IFN phenotypes originally attributed to pp65 appear to be due to the actions of other viral gene products such as IE2, new roles for pp65 are continually being revealed, such as the interaction between pp65 and activators of the stimulator of interferon genes (STING), an IFN inducer. Signaling via STING and the dsDNA sensor cGAS has emerged as an important cellular defense against viral infection [33]. A novel binding interaction between pp65 and cGAS was recently identified as a mechanism through which pp65 inhibits the release of cGAMP, thereby preventing STING recruitment to cGAS and impeding the expression of IFNβ [34]. STING signaling is also reported to be inhibited by pp65 via interaction with the upstream STING activator interferon gamma-inducible protein 16 (IFI16). Evidence suggests that pp65 binds to IFI16 and occludes the pyrin domains required for IFI16 oligomerization in the nucleus, preventing STING activation and anti-viral cytokine expression [35]. Contributing to this picture, pp65 has also been shown to form a complex with UL97 [36], a conserved herpesviral modulator of IFN signaling that downregulates IFN secretion via inhibition of IRF3 [37], and may have further direct or indirect effects on IFI16 activation mediated through this interaction. These recent findings underscore the extent to which pp65’s interactions with IFN signaling are continuing to be elucidated.

Evidence has emerged implicating several other tegument proteins in innate immune modulatory activities. These include the U_L_23, U_L_26, and pp71 proteins. The pp71 tegument protein, encoded by the U_L_82 gene, shares significant homology with its neighboring HCMV gene product pp65 [38,39] and has been identified as a key activator of the HCMV major immediate-early promoter (MIEP) [40]. Known to be required for high-titer viral replication [41], pp71 has been discovered to provide an integral defense against silencing of the viral MIEP by the host innate immune effector Daxx [42]. Through direct binding, pp71 functions to disrupt complex formation between Daxx and other transcriptional repressors such as ATRX [43], as well as target Daxx itself for proteasomal degradation [44]. Underscoring the relevance of this interaction is the observation that ablation of pp71 results in attenuated expression of HCMV immediate-early genes and impaired lytic replication, which can be rescued via inhibition of Daxx [41,45]. pp71 has also recently been found to bind STING and prevent the formation of complexes required for its translocation, sequestering it away from the nucleus [46]. Further, RNAi-mediated knockdown or gene knockout of pp71 increases host anti-viral gene expression, further emphasizing the importance of pp71 for attenuating innate immunity during infection [46].

Tegument proteins have also been shown to modulate the Type II IFN response to infection. Type II signaling, induced by IFNγ, is heavily dependent on STAT1 transcription factor homodimers that bind and activate transcription at promoters containing gamma interferon activation sequences (GAS) [47]. Recent work using U_L_23-deficient HCMV mutants shows that Type II IFN gene targets are upregulated during infection in the absence of U_L_23. These mutants are also more sensitive to challenge with IFNγ [48]. A putative binding interaction between U_L_23 and the STAT effector molecule N-myc interactor (NMI), identified via yeast two-hybrid screening, is hypothesized to prevent the proper activation and translocation of the STAT1 homodimers required for Type II IFN signaling [48]. These early results provide insight into HCMV’s modulation of Type II IFN, which is less well-characterized than its interplay with Type I signaling.

Complex relationships between tegument proteins and individual interferon stimulated genes are also currently being elucidated. The ubiquitin-like modifier ISG15 is a Type I IFN target that is covalently bound to target molecules, thereby altering their function (reviewed in [49]). Multiple reports have implicated ISGylation as an anti-viral defense mechanism that is triggered early during HCMV infection via cGAS-STING viral DNA sensing and restricts HCMV replication [50,51]. U_L_26 was recently found to interact with ISG15 as well as multiple enzymes involved in the activation and ligation of ISG15 to target proteins [51]. Interestingly, some of these ISG15 enzymes have other potentially relevant roles in the immune response outside of their ISG15-proximal functions, such as UBP43, which ligates ISG15 to target proteins but also binds IFNAR2 and occludes its interaction with JAK1 to downregulate IFN signaling [52]. During infection in the absence of immediate-early protein 1 (IE1), U_L_26 appears to play a role in suppressing the accumulation of ISGylated proteins [51]. Notably, U_L_26 itself is subject to ISG15 modification [51]; however, many questions remain about how the interactions between U_L_26 and the ISG15-machinery contribute to viral infection.

In addition to proteins involved in cytoplasmic DNA sensing, e.g., IFI16, ZBP1, and cGAS, the host cell also encodes proteins that sense cytoplasmic dsRNA and activate similar responses, with one of the most prominent being PKR (reviewed in [53]). PKR is an ISG with an N-terminal dsRNA-binding domain that can be activated by the presence of dsRNA as well as by a host of other stimuli including oxidative stress, cytokines, and other cellular kinases [54]. Active PKR functions by homodimerizing and autophosphorylating itself to enable a binding interaction between its C-terminal domain and the eukaryotic transcription initiation factor eIF2α, globally repressing transcription of both viral and cellular genes by interfering with the formation of the eIF2α-tRNAMet-GTP transcription initiation complex [55,56]. PKR signaling can result in a diverse array of immune outcomes including upregulated Type I IFN signaling [57] as well as increased NFκB pathway activity [58]. HCMV encodes two immediate-early gene products that specifically target PKR as a means of downregulating the IFN response and maintaining high levels of viral gene transcription during infection: IRS1 and TRS1.

Co-infection of HCMV was found to be sufficient to rescue protein translation of a Vaccinia virus mutant (VVΔE3L) that is sensitive to PKR activity [59], which ultimately led to the identification of IRS1 and TRS1 as key PKR modulatory factors [60]. Further investigation into TRS1 found that two distinct regions of this protein modulate PKR in separable fashions, with the C-terminal region capable of directly binding dsRNA and presumably preventing it from binding PKR and other dsRNA sensors, and the N-terminal region binding PKR directly and sequestering it in the nucleus to prevent its activation and downstream signaling [61,62,63]. Interestingly, both regions of TSR1 are required to fully rescue VVΔE3L-PKR related phenotypes, which include viral replication and host cell range [61], though more recent results suggest that the N-terminal region is responsible for the majority of the PKR inhibition phenotype [64].

To assess the contribution of these proteins to overall HCMV replication, mutants containing deletions of IRS1 and TRS1 both individually and in tandem were used to show that loss of either gene product in isolation did not strongly affect viral growth, but infection with an IRS1/TRS1 double deletion mutant resulted in an extreme reduction in protein synthesis and failure to replicate in primary human fibroblasts [65]. A follow up study showed that this pattern of growth correlated to levels of PKR activation as well as that viruses harboring individual mutations of IRS1 and TRS1 maintained the ability to suppress PKR activation during infection, whereas siRNA silencing of PKR rescued viral growth in the context of simultaneous loss of IRS1 and TRS1 [66]. These results indicate that modulation of IFN signaling by IRS1 and TRS1 directly contributes to infectious outcomes. As future inquiries continue to dissect the different functions of IRS1 and TRS1, it will be important to elucidate the nuances of IRS1 function, which has received less scrutiny to date than TRS1.

### 2.2. HCMV Immediate-Early Genes (IE) and Interferon Modulation

Immunomodulatory tegument proteins enjoy a temporal advantage over viral genes that must be expressed de novo during infection as there is no delay between the onset of infection and their time of action. However, studies investigating the role of de novo viral protein synthesis in influencing the interferon response to HCMV infection using UV-inactivated virus [30,32] and cycloheximide treatment [67] have found that, in addition to the suite of tegument-delivered viral proteins, newly synthesized viral proteins are also required for wild-type interferon pathway modulation. Recently identified as an HCMV-encoded regulator of ISG15 and ISGylation, IE1 (IE72, pU_L_123) was observed to strongly inhibit ISG15 transcription levels during viral infection and enable viral evasion of the ISGylation response [51]. This discovery is just the latest in a collection of work pointing to IE1’s ability to modulate Type I IFN signaling. Initial studies found that a recombinant virus lacking IE1 was vulnerable to Type I IFN treatment [68]. Further work revealed that IE1 binds to STAT2 via its carboxy-terminal acidic domain, which is also required for high-titer viral replication, and that this interaction interferes with the ability of STAT2-IFN-stimulated gene factor 3 (STAT2-ISGF3) complexes to bind to interferon stimulated response elements (ISRE) and increase the transcription of Type I IFN targets [69]. Interestingly, IE1 appears to prevent STAT2-ISGF3 complex loading onto ISRE sites without altering the abundance, phosphorylation, or complex formation ability of STAT2, relying on a mechanism of interference that has yet to be fully described [68,69]. Additionally noteworthy is that SUMOylation of IE1’s acidic domain has been shown to inhibit the IE1-STAT2 interaction and mitigate the impact of IE1 on IFN target gene expression, indicating that the cell has evolved countermeasures to respond to the activities of IE1 during infection [69].

In addition to modulation of the Type I pathway, IE1 expression has been reported to alter Type II IFN signaling. Multiple studies show that endogenous expression of IE1 in human fibroblasts is sufficient to shift the host transcriptional profile to one resembling IFNγ-treated cells [70,71] This transcriptional response was shown to occur independently of IFNγ [70], and IE1 does not appear to directly interact with STAT1, the major Type II transcription factor [72]. Further investigation led to a model in which IE1 binds STAT3 and sequesters it in the nucleus, preventing its phosphorylation [71]. In the absence of cytoplasmic STAT3, STAT1 phosphorylation and activation by the cytoplasmic kinase JAK1 is increased, resulting in higher levels of phosphorylated STAT1 localizing to the nucleus and inducing transcription of Type II IFN target genes [70]. However, other recent findings indicate that endogenous IE1 is capable of reducing STAT1 homodimer binding to Type II GAS promoter elements, complicating the story [72]. Collectively, it is clear that many questions about the interplay between IE1 and Type II IFN signaling remain to be resolved.

IE2 (IE86, pU_L_122) has also been closely linked with IFN modulation during infection. IFNβ production during infection is strongly inhibited by the presence of IE2 [73], which has been found to suppress IFNβ levels by binding NFκB and preventing its interaction with NFκB sites located on the IFNβ promoter, thereby inhibiting transcription [32]. Notably, a more current analysis has broadened the scope of potential interactions between IE2 and Type I IFN by showing that IE2 also targets the IFN activating molecule STING for proteasomal degradation and attenuates STING-induced transcription of IFNβ, potentially through two distinct mechanisms [74]. While many questions remain, evidence of modulation of interferon signaling by IE1 and IE2 strongly supports the notion that targeting interferon signaling at immediate-early times of infection is critical for successful HCMV infection and persistence.

### 2.3. HCMV-Mediated Modulation of Interferon at Later Times of Infection

The unique short region of the HCMV genome contains a series of adjacent genes with limited sequence similarity, U_S_2–U_S_11, that encode a set of glycoproteins implicated in a variety of activities including immune modulation [75,76]. U_S_9 is expressed with early kinetics during viral infection and localizes to the mitochondria and endoplasmic reticulum [77], where it appears to modulate Type I IFN signaling and IFNβ production through two distinct interactions with the MAVS and STING adaptor proteins [78]. MAVS and STING both function similarly as adaptors that recruit the TBK1 kinase with its substrate, IRF3, which is then activated and localized to the nucleus to upregulate ISG transcription [78]. Expression of U_S_9 in cells results in a strong downregulation of IRF3 activation and nuclear accumulation [78]. U_S_9 appears to achieve this effect by damaging the cell’s mitochondria and preventing its retention of MAVS, as well as by directly binding STING and preventing its dimerization and downstream activation of IRF3 [78]. As a non-tegument HCMV protein expressed with early kinetics, U_S_9 is of particular interest because most HCMV-encoded IFN modulatory proteins discovered to date are either delivered with the tegument or expressed immediately upon infection. In contrast, U_S_9 is scarcely detectable within the cell prior to 6 hpi and reaches its highest level of accumulation around 48 hpi [23], appearing to be exclusively dedicated to protecting and enabling the later phases of the viral life cycle.

As discussed above, the identification of pp71 as a cGAS-STING interactor was achieved through the use of an HCMV gene expression library containing 131 constructs each encoding a unique HCMV protein [46]. Another HCMV gene product that emerged from this screen was U_L_31, a true-late protein required for wild-type viral growth [79,80] that was found to be capable of binding both viral DNA and host cell cGAS via its N- and C-terminal regions, respectively. U_L_31’s high binding affinity for cGAS, but not for DNA, suggests a non-competitive mechanism of action wherein U_L_31 preferentially binds cGAS in a manner that dissociates DNA from the molecule. U_L_31 fails to inhibit cGAMP induction of Type I ISGs but is capable of inhibiting the interferon-associated gene transcription stimulated by both HCMV infection and dsDNA, signifying that this key interaction with cGAS is critical for modulating downstream immune signaling [81]. Further, knockdown of cGAS is capable of rescuing the growth defect of U_L_31-deficient viral infection, indicating that the U_L_31/cGAS interaction has implications for overall viral growth [81]. Notably, U_L_31-mediated inhibition of cGAS appears to have a broad immune footprint, affecting multiple immune signaling archetypes, as infection with a U_L_31-deficient HCMV mutant strongly induced both ISG’s and NFκB target genes, and plasmid overexpression of U_L_31 in fibroblasts inhibited the activation of both ISRE-containing and NFκB reporter elements [81].

Another upstream inducer of STING, IFI16, is targeted for inhibition by the HCMV early-late gene U_L_97. Conserved among herpesviruses, U_L_97 is a viral kinase that has been shown to bind and phosphorylate IFI16, inducing its relocalization out of the nucleus of infected cells into the cytoplasm, where it is prevented from inducing an IFN response [82]. Nuclear retention of IFI16 can be rescued by treatment with an inhibitor that reduces U_L_97 kinase function or by removing the U_L_97 reading frame from the HCMV genome, further establishing this link between U_L_97 and IFI16 export [82].

### 2.4. Modulation of Interferon during Latency

Work using an IRS1 and TRS1 double mutant (ΔIRS1/ΔTRS1) to investigate PKR modulation during infection was instrumental in uncovering another HCMV gene product that alters the host cell’s ability to sense viral dsRNA: ORF94 (U_L_126a). Oligoadenylate synthetase (OAS) proteins are dsRNA-binding enzymes that activate cellular RNases in response to dsRNA detection, degrading host and viral RNA and down regulating overall rates of protein synthesis (reviewed in [83]). HCMV infection institutes a block to OAS signaling, and it was observed that while HCMV-encoded TRS1 and IRS1 are capable of downregulating this pathway in certain contexts [60], a mutant HCMV lacking both of these open reading frames still inhibited OAS activation [65]. HCMV ORF94 was identified as a potential candidate that may be mediating this OAS block by inhibiting the expression of OAS1 in multiple contexts, including during productive infection and in the face of interferon stimulation [84]. Notably, HCMV ORF94 is a latency-associated ORF encoded by transcripts expressed during latent infection [85]. While extremely intriguing, it still remains to be seen how ORF94 and its modulation of the IFN response contributes to latency establishment, maintenance, or reactivation.

## 3. HCMV Modulation of NFκB Signaling

**Figure 2:**
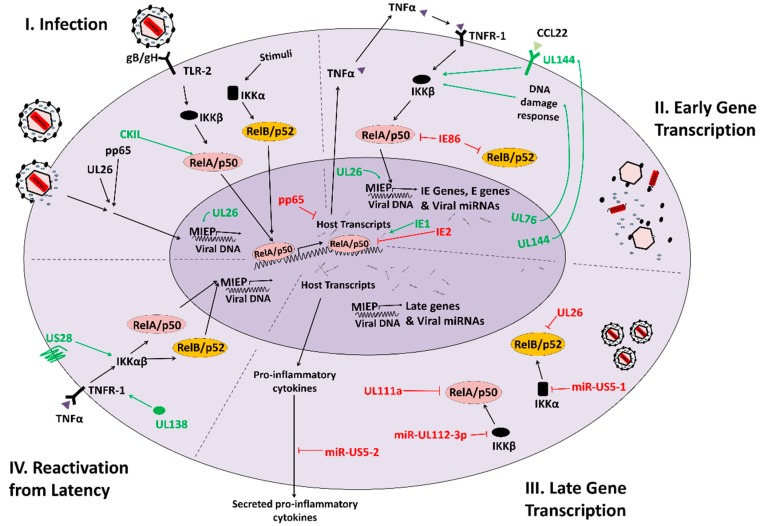
HCMV-mediated modulation of NFκB signaling

The NFκB signaling network regulates a wide variety of pro-inflammatory processes that ultimately shape innate and adaptive immune responses via transcriptional regulation of numerous NFκB responsive genes. NFκB signaling can be activated by a myriad of inducers including infectious agents, paracrine signaling factors, or environmental stress [86]. Highlighting its importance as a defense against infection, a wide variety of evolutionarily diverse viruses including Human Immunodeficiency Virus-1 (HIV-1), HCMV, Herpes Simplex Virus-1 (HSV-1), and Epstein Barr Virus (EBV) have evolved mechanisms to modulate NFκB signaling [87]. Numerous interactions between HCMV and NFκB signaling have been described over the years (Figure 2, and reviewed in [87,88]). HCMV gene products have been shown to both inhibit NFκB signaling and activate facets of the NFκB pathway to support lytic replication or induce reactivation from latency [89,90], suggesting a nuanced relationship. This highlights the reality that NFκB signaling is not simply binary, but rather that NFκB endpoint signaling is capable of multiple distinct transcriptional landscapes depending on specific upstream stimuli. The relationship between HCMV and NFκB is shaped by multiple *HCMV* gene products, including both proteins and miRNAs, which serve to modulate various aspects of NFκB signaling. Less clear are how the various facets of HCMV-mediated modulation of NFκB contribute to the variety of biological contexts of HCMV infection, including viral persistence, shedding, latency, reactivation, tropism, and pathogenesis.

Most effectors of the NFκB response converge upon the activation of a serine-specific IκB kinase (IKK) complex comprised of different combinations of three distinct subunits: IKKα, IKKβ and IKKγ/NEMO (Figure 2). Canonical NFκB signaling relies on a tripartite complex consisting of one of each IKK subunit, while non-canonical NFκB functions through an IKKα dimer [86]. The mechanisms through which the cytoplasmic NFκB subunit complexes are activated represent a major difference between these two sub-pathways (Figure 2). In canonical NFκB signaling, an IKK complex containing NEMO phosphorylates the repressor protein IκB, marking it for ubiquitination and degradation. IκB represses the canonical NFκB transcription factors p50 and p65 (RelA) in the cytoplasm, which are freed upon IκB phosphorylation/degradation, resulting in their nuclear translocation and subsequent transcriptional activation of NFκB targets [80]. During non-canonical NFκB signaling an IKKα homodimer acts as the kinase that phosphorylates the C-terminus of p100, which resides in an inhibitory complex with RelB in the cytoplasm. This phosphorylation of p100 results in its processing to p52, which in complex with RelB, can enter the nucleus to modulate NFκB target transcription [91] (Figure 2). 

During HCMV infection of fibroblasts NFκB activation appears to follow a specific sequence in which the pathway is active early in infection, but is then repressed from middle to late time points of the viral life cycle. At the earliest time of infection, i.e. envelope fusion, the HCMV glycoproteins B and H (gB/gH), encoded by U_L_55 and U_L_75, respectively, bind to Toll-like receptor 2 (TLR-2) on the surface of the cell and induce a canonical NFκB signaling cascade resulting in the excretion of pro-inflammatory and anti-viral cytokines [92]. This immediate enhancement of NFκB activation appears to be pro-viral, as HCMV’s MIEP possesses NFκB binding motifs [93] that facilitate the expression of IE genes, an effect that can be enhanced by TNFα stimulation [94]. Further evidence supporting a pro-viral aspect of early NFκB activation for infection include the findings that dominant-negative constructs targeting key NFκB constituents such as IKKα, IKKβ, and IκBα reduce MIEP activation [95]. These findings suggest that NFκB activation at early times is important for optimal transactivation of the MIEP. However, the utilization of HCMV mutants lacking the NFκB motifs in the MIEP did not result in significant attenuation of infection or IE gene product accumulation [96], suggesting that additional host transcription factor binding motifs present in the MIEP, including CREB (cAMP response element binding) and ATF (activating transcription factor) sites [97], may be sufficient to activate IE gene transcription even in the absence of p65/p50 binding. This remains an unresolved issue, and further inquiries have suggested that this nuanced interaction between viral transcription and NFκB signaling can be influenced by numerous factors including the host cell’s progression through the cell cycle [89], the adaptation of HCMV strains to laboratory passage conditions [98], and host cell lineage [99].

### 3.1. HCMV Tegument Proteins and NFκB Modulation

Concurrent with envelope fusion, the virion releases viral tegument proteins into the cytoplasm which disseminate and begin to modulate a number of cellular pathways. The pp65 protein, as discussed above, likely plays a role in blocking the host IFN response during early infection that is not yet fully understood. In addition to this IFN modulatory role, infection with a pp65-deficient mutant HCMV increases the accumulation of NFκB target genes and induces the nuclear binding activity of NFκB transcription factors [31], suggesting an important contribution to NFκB regulation. Mechanistically, however, it is unclear how pp65 modulates NFκB activity. Further, the extent to which pp65’s modulation of IFN and NFκB signaling might be functionally related is uncertain.

The U_L_26 protein is also delivered with the tegument and has been shown to antagonize NFκB activity. A U_L_26 deletion mutant virus is severely attenuated, and U_L_26 has been shown to be necessary to inhibit IKK complex phosphorylation and RelB translocation during infection [100,101]. Further, expression of U_L_26 by itself is sufficient to block TNFα-mediated NFκB activation [100,101]. However, despite its presence in the tegument, U_L_26 does not block the activation of NFκB at the earliest times of infection. U_L_26 is thought to have a stronger role in the attenuation of NFκB activity that occurs later during infection, when U_L_26 localizes to the cytoplasm, as opposed to early times when it localizes in the nucleus [102]. However, the possibility of an interaction between U_L_26 and NFκB at early times during infection cannot be ruled out, as U_L_26-deficient virus is more sensitive to challenge with TNFα [100]. Notably, U_L_26 is capable of inhibiting IKK complex activation in the face of diverse upstream stimuli including TNFα signaling and Sendai virus infection, suggesting that it is acting at the level of the IKK complex, where these signaling cascades converge [100], but the exact mechanism of U_L_26’s inhibition of NFκB signaling remains to be elucidated

HCMV tegument proteins are also capable of inducing pro-viral NFκB signaling. U_L_76, a tegument-associated endonuclease, is reported to activate the canonical NFκB pathway through the DNA damage response and induce IL-8 production [103]. This U_L_76-mediated increase in IL-8 production was shown to be dependent on the cellular kinases Ataxia-telangiectasia mutated (ATM) and IKKβ; however, ablation of ATM expression in cells or of the key endonuclease motif amino acids present in U_L_76 failed to completely restore IL-8 production back to wild-type levels during infection [103], implying that additional aspects of NFκB signaling might be contributing to this phenotype. A U_L_76 deletion mutant has a significant growth defect [104], but the contributions of increased IL-8 production to this attenuation are not clear.

HCMV virions have been found to incorporate cellular mRNAs and proteins [27,105]. This raises the possibility that, in addition to viral factors, virion-associated cellular factors could also be modulating NFκB signaling. One such example is the virion packaging of casein kinase II (CKII), which has been found in the virion tegument and is reported to activate NFκB signaling through phosphorylation of the IκB repressor, thereby releasing the associated NFκB subunits to localize to the nucleus and induce NFκB-dependent transcription [106]. The extent to which cellularly-derived, virion-associated NFκB modulators contribute to the various facets of HCMV infection is still largely not known.

### 3.2. HCMV Immediate-Early Genes and NFκB Modulation

The IE proteins expressed upon MIEP stimulation interact with the NFκB pathway in diverse ways. IE1, a promiscuous transactivator of NFκB pathway constituents and their downstream targets during infection, has been implicated in the upregulation of p65, IL-6, TNF-α and IL-8 as well as the induction of p52/RelB heterodimer binding activity in the nucleus [88]. At immediate-early times, the virus also produces U_L_144, a TNF-receptor-like transmembrane receptor with immediate-early expression kinetics [107], which activates expression of the immune cytokine CCL22 [108]. Mechanistically, U_L_144 complexes with TRAF6 in perinuclear regions of the cell to enable NFκB transcription factor translocation and binding, and siRNA targeting U_L_144, TRAF6, or NFκB all ablate the downstream CCL22 expression induced by infection [108]. The CCL22 cytokine has been noted to play a chemoattractant role in recruiting Th2 and regulatory T-cells (Tregs) to mediate adaptive immune responses [108]. The U_L_144 open reading frame is lost in extensively laboratory passaged strains of HCMV, potentially due to its activation of NFκB signaling, which could be a detriment to viral fitness during in vitro fibroblast infection [109,110].

In addition to immediate-early expression of the NFκB agonists IE1 and U_L_144, IE2 inhibits host NFκB signaling at all points during HCMV infection through a still-controversial model: either by blocking NFκB subunit dimer interactions or preventing subunit interactions with specific NFκB target promoters, e.g., IL-6 [32,111]. Interestingly, the antagonistic effects of IE2 do not prevent U_L_144 from inducing NFκB [112], which highlights the specificity with which HCMV is able to tailor NFκB signaling. Collectively the data suggest that at early points of infection the virus seems to be operating within an optimal pro-inflammatory signaling window, with just enough NFκB transcription factor binding to transactivate the viral MIEP, but still staying below a threshold that might trigger a broader anti-viral immune response.

### 3.3. HCMV-Mediated Modulation of NFκB at Later Times of Infection

At some point during the transition from the immediate-early stages of infection, where active NFκB signaling is observed, to the later stages of infection, the viral actions towards NFκB become much more inhibitory. This transition coincides with the increased expression of multiple HCMV genes that antagonize NFκB activity. One such gene is U_L_111a, also known as cmvIL-10 for its functional similarity to the cellular cytokine IL-10. cmvIL-10 shares only approximately 27% homology with host IL-10, but binds as a homodimer to the human IL-10 receptor and blocks NFκB signaling by preventing IκBα degradation in a similar manner to IL-10 [113,114,115]. In addition, cmvIL-10 also has a significant immunosuppressive effect on interferon signaling. In peripheral blood mononuclear cells (PBMCs), TLR-agonist treated media from wild-type AD169 infection is sufficient to inhibit the production of IFNα when compared to similarly treated media from a cmvIL-10 knockout virus [116]. Further, cmvIL-10 alone is sufficient to do the same in plasmacytoid DCs [117]. The mechanism behind these inhibitions appears to involve activation of STAT3 [118], but is still poorly understood. It remains unknown if the suppressive effects of cmvIL-10 on IFN and NFκB signaling are separable. Studies of the cmvIL-10/interferon signaling axis suggest that the molecule may be acting in a paracrine manner to activate defenses in uninfected cells, which could represent a promising avenue of further inquiry.

### 3.4. HCMV miRNAs and NFκB Modulation

Viral miRNAs represent additional tools that HCMV employs to undermine host cell defenses at later points of infection. HCMV encodes 26 miRNAs, each approximately 22nt in length, that have been implicated in modulating a wide variety of cellular pathways and processes including vesicle transport, cytokine secretion, immune signaling, and progression through the cell cycle, (reviewed in [119] and [120]). Throughout the course of infection, beginning with immediate-early gene expression, HCMV miRNAs begin to accumulate, becoming abundant by late infection [119,121]. HCMV miR-U_S_5-1 and miRU_L_112-3p have been shown to prevent NFκB cytokine signaling by specifically downregulating the expression of the key kinases IKKα and IKKβ [122]. In addition to blocking the NFκB signal relay set off by cytokine detection, miR-U_S_5-2 is able to block the infected cells’ secretion of cytokines [123], thereby ceasing the positive feedback loop of NFκB activation and ultimately returning the pathway to its initial inactive state. During latent infection, miR-U_L_148D is one of the most highly expressed miRNAs, and has been demonstrated to block NFκB upstream adapters and repress IL-6 production [124], thus allowing the infected cell to evade host immunity.

### 3.5. Latency-Associated NFκB Modulators

A hallmark of all herpesviruses is the ability to enter latency, persisting with limited lytic viral replication in the face of a primed immune response and capable of reactivation during times of stress or immunosuppression, resulting in viral dissemination and potential pathologies. The signals that reactivate HCMV from latency remain incompletely understood, but immunosuppression and inflammation are thought to play major roles (reviewed in [125]). Consistent with this view, reports indicate that viral genes activate NFκB during reactivation [126], and, further, NFκB activation has been linked to HCMV reactivation via NFκB subunit enhancement of MIEP expression [127,128]. The viral chemokine receptor U_S_28, expressed with early kinetics during lytic infection, is one of the few viral proteins expressed during latency as demonstrated in latently infected THP-1 monocytes [128]. U_S_28 has been implicated in activating the MIEP through NFκB signaling [126]; it is possible that during latent expression, U_S_28 activates the MIEP and aids in reactivation from latency. U_S_28 induces constitutive NFκB activation through its interaction with the Gq/11 G protein, which mediates the release of Gβγ subunits that induce downstream NFκB activity [129]. Although in general U_S_28 has appeared to stimulate NFκB activity, recent work suggests that U_S_28 attenuates multiple cell signaling pathways including NFκB, which is required to maintain latency as mutants lacking U_S_28 return to their lytic phase and infected cells are subsequently targeted by T-cells [130]. It is clear that U_S_28 plays a complicated role in the HCMV life cycle; and, like other viral factors, possesses more than one role that may seem counterintuitive, but that are likely important in different infectious contexts.

Another viral protein expressed during latency, U_L_138, acts by activating and stabilizing the cell surface expression of TNFR1 [131,132,133]. Interestingly, while U_L_138 appears to promote the sensitivity of latently infected cells to TNFα, reporter assays show that U_L_138 strongly represses MIEP transactivation [134] and ChIP (chromatin-immunoprecipitation) assays suggest it prevents cellular demethylases from interacting with the MIEP [135], leading to the conclusion that this protein is also playing a central role in maintaining HCMV’s latent state. These results represent some of the first forays into exploring the immunomodulatory potential of HCMV latency-associated genes, and the data suggest that there is a complex interplay between pro- and anti-viral manipulations of immune signaling as the virus maintains latent infection despite silencing much of its transcription.

## 4. Conclusions and Future Perspectives

Reports over the past 25 years have made it abundantly clear that HCMV devotes substantial genetic resources towards manipulation of interferon and NFκB signaling. The list of involved genes is large and still growing, with a variety of HCMV gene products playing modulatory roles in various contexts. While a multitude of questions remain, certain themes and patterns have emerged. For example, with respect to IFN signaling, while some ISGs such as viperin and Cox2 (reviewed in [12]) appear to be co-opted by the virus for pro-viral activities, almost all HCMV gene products that have been identified to play a role in IFN signaling serve to attenuate this host cell response to support robust infection, suggesting that HCMV goes to great lengths to inhibit IFN signaling to enable infection.

In contrast, the literature regarding HCMV’s modulation of NFκB suggests a more complex picture. NFκB activation during lytic infection of fibroblasts appears to be biphasic with a pro-inflammatory, pro-viral NFκB signaling environment instituted during the earliest times of infection, which subsequently shifts to broadly inhibitory of NFκB at later time points of infection. As discussed above, current data suggest a model in which early NFκB activation supports infection through increased MIEP transcription, whereas later NFκB inhibition prevents the secretion of NFκB-regulated anti-viral factors, e.g., cytokines. However, many questions remain. For one, the sheer number of HCMV gene products that modulate NFκB inflammatory signaling in diverse ways suggests a more nuanced story. It seems likely that these NFκB modulatory gene products work in different infectious contexts to support varied aspects of viral infection, including robust lytic infection, the establishment of latency, and reactivation from the latent state. In this regard, the activities of NFκB-modulatory viral proteins will likely be sensitive to a variety of cellular states including cell type, differentiation status, and the inflammatory environment.

A typical in vivo infection progresses from the mucosal epithelial cells to responding immune cells, with subsequent seeding of lymph nodes and infection of progenitor cells that will serve as latency reservoirs, followed by some level of either subclinical or pathogenic reactivation back in the mucosal epithelia. We propose that cell-type and context specific HCMV-mediated modulation of NFκB will be critical at each step of this process. Furthering our understanding of how the relationship between HCMV and NFκB shapes viral biology and pathogenesis will require elucidating how specific mechanisms of HCMV-mediated modulation of NFκB contribute to key events during the in vivo viral life cycle in physiologically relevant cell types. While this will be challenging given the limitations of our current in vivo and in vitro models, recent developments in humanized mice, as well as explant and organoid culture techniques will yield exciting, novel opportunities to address these issues. The field has made substantial progress, likely identifying the majority of the viral players involved in NFκB modulation. It is time to turn our attention to identifying how these viral NFκB modulators contribute to various infectious contexts, and to developing the tools and experimental systems required to do so. Given the importance of these interactions to infectious outcomes, further elucidating how they contribute to infection will likely provide novel avenues to limit HCMV-associated pathogenesis.
